# Immediate post-extraction implants placed in acute periapical infected sites with immediate prosthetic provisionalization: a 1-year prospective cohort study

**DOI:** 10.4317/medoral.23708

**Published:** 2020-08-27

**Authors:** Daniel Muñoz-Cámara, Osmundo Gilbel-Del Águila, Guillermo Pardo-Zamora, Fabio Camacho-Alonso

**Affiliations:** 1 DDS, PhD. Department of Prosthetic Dentistry, University of Murcia, Spain; 2 DDS, PhD. In private dental practice, Murcia, Spain; 3 DDS, PhD. Department of Integral Dentistry, University of Murcia, Spain; 4 DDS, PhD. Department of Oral Surgery, University of Murcia, Spain

## Abstract

**Background:**

Few studies have reported the outcomes of immediate placement at infected post-extraction sites. The aim of this study was to compare clinical and radiological outcomes of immediately placed implants with immediate prosthetic provisionalization in sockets with or without acute periapical pathology.

**Material and Methods:**

A total of 100 patients with immediately placed implants with immediate provisionalization and 1- year of follow up were included (50 patients with acute periapical pathology and a control group of 50 patients without acute periapical pathology). Clinical parameters (bleeding on probing, buccal keratinized mucosa width, clinical recession, and probing depth) and radiological parameters (distance from implant shoulder to first point of bone-to-implant contact [IS-BIC]) were assessed.

**Results:**

Clinical parameters showed no significant differences between the study and control groups after 1-year follow up (*p*>0.05). IS-BIC presented the following values: 0.35 ± 0.51 mm (study group) and 0.15 ± 0.87 mm (control), without significant differences between the groups (*p*=0.160). None of the 50 radiographs of immediate implants placed in sockets with periapical pathology revealed retrograde peri-implantitis.

**Conclusions:**

Immediate placement of implants with immediate prosthetic provisionalization at sites with acute periapical pathology can be a successful treatment modality for at least 1-year.

** Key words:**Immediate dental implants, immediate prosthetic provisionalization, periapical pathology.

## Introduction

According to retrospective and prospective studies, immediate implant placement in a fresh extraction socket has become a predicTable technique with reported success rates of over 93% (1-4). The technique takes advantage of the organism’s regenerative potential following extractionand helps to preserve the volume of both bone and soft tissue. In this way, it reduces the crestal bone loss that occurs after extraction and bone healing (5). In addition to the high success rates achieved with the technique, the reduction in treatment time together with patients’ high level of satisfaction with esthetics has made immediate implant placement a routine procedure in many dental clinics (6,7).

During immediate implant placement, preservation of crestal bone is important as this influences the formation of papillae and the attainment of favorable esthetics (8). This positive association between crestal bone preservation and attaining favorable soft tissues makes the technique an attractive therapeutic option, affirmed both in animal models and clinical practice (9,10). Ensuring that bone and peri-implant soft tissues are sTable is a basic criterion of dental implant success (11).

The preservation of hard and soft tissues with immediate post-extraction implants can be enhanced by immediate prosthetic provisionalization, which offers the patient a range of additional advantages that are social, psychological, functional and esthetic (12,13). The great advantage of immediate implant provisionalization is its capacity to minimize the loss of soft tissue volume, making it a good therapeutic option that can be considered whenever the case’s occlusal situation, and the implant’s primary stability will permit (14).

Implant placement at sites presenting periapical or periodontal infection remains a subject of debate within the field of implant dentistry. Few published studies have evaluated the efficacy of immediate implants in areas presenting pathology, a procedure that would simplify implant-based treatments by reducing the number of surgical steps, providing the risk of implant loss can be eliminated (15). But various studies advise against this (16,17), arguing that retrograde peri-implantitis could be caused by the presence of alveolar granulomatous tissue remnants with periapical pathology, which were not eliminated correctly at the moment of extraction and immediate implant placement (18). But new research has shown that immediate implants placed in alveolae with periapical pathology in animal models achieve the same success rates as those placed in alveolae without pathology (19-22).

Five prospective controlled clinical trials have been published assessing clinical and radiological variables for immediate post-extraction implants placed in patients with periapical pathology (23-27). It was found that success rates were similar between cases with and without periapical pathology; three works with 1-year follow-up (23,24,27) and two works with 3- and 4-year follow-up periods respectively (25,26). To date, only one trial has been conducted of immediate implant placement in alveolae with periapical pathology that has also performed immediate prosthetic provisionalization, but this work lacked a control group of cases without periapical pathology (28).

So, the aim of the present prospective cohort study was to compare the clinical and radiological outcomes of immediate post-extraction implants with immediate prosthetic provisionalization in sites with and without peri-apical pathology with a 1-year follow-up.

## Material and Methods

- Recruitment and patient characteristics

The study protocol was approved by the University of Murcia (Spain) Ethics Committee (1547/2017) and was carried out between May 2017 and December 2018 at two centers: the University Dental Clinic (University of Murcia, Murcia, Spain) and a private dental clinic. A prospective cohort study design was used, in accordance with Strengthening the Reporting of Observational Studies in Epidemiology Guidelines (STROBE) (29). Inclusion criteria were as follows: patient aged over 18 years, need for dental extraction (with acute periapical pathology: failed endodontic treatment, active periapical periodontitis, granuloma and pus or infected root fracture; or no infection), absence of medical contra-indications for oral surgical procedures (ASA I / II), presence of indications for immediate implant placement, patient willing to provide informed consent to take part. Exclusion criteria were: presence of some disease, condition, or medication that could compromise healing or osteointegration (diabetes mellitus, bisphosphonate administration, or severe osteoporosis); patient presenting complete loss of vestibular or lingual cortex; presence of severe mental disorder; and patients who had received radiotherapy of the head and neck during the previous 18 months.

None of the patients who fulfilled the inclusion criteria and were invited to take part in the trial consecutively refused to do so. The total sample was 100 patients (50 with acute periapical pathology and 50 without acute periapical pathology) who were treated according to guidelines established by the declaration of Helsinki for medical research involving human subjects. Fifty patients comprised the test group (presenting acute periapical pathology observed in a presurgical examination using cone beam computed tomography; and presence of typical acute infection signs in the clinical evaluation: fistula with or without suppuration, pain, swelling, suppuration from the gingival sulcus, tooth mobility, or a combination of these findings), and fifty patients without acute periapical pathology formed the control group.

Before surgery, patients’ sociodemographic data were registered, as well as their status regarding smoking and alcohol consumption, and their complete medical histories. Variables relating to oral hygiene maintenance were also registered: frequency of tooth brushing, and O’Leary *et al* *et al*., full-mouth plaque index (30).

- Surgical procedure

All patients carried out the following pre-operative regime: 0.12% chlorhexidine mouthwashes (three times a day for 4 days before and 7 days after surgery) and 875/125 mg amoxicillin/clavulanic acid (three times a day for 4 days before and 7 days after surgery; in cases of penicillin allergy, 300 mg clindamycin was prescribed every 8 hours). All surgery was performed by the same clinician under local anesthesia.

**Figure 1:**
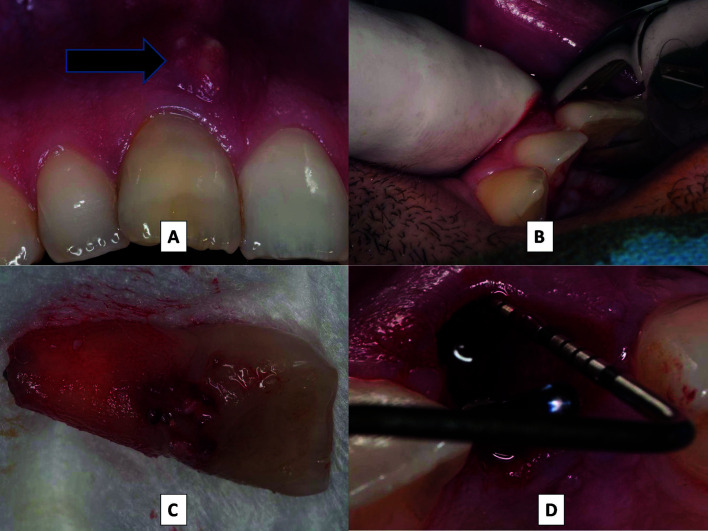
A: Central upper right incisor with acute infection (failed endodontic treatment) with presence of intraoral fistula (black arrow). B: Careful extraction causing minimal trauma. C: Extracted tooth. D: Checking integrity of vestibular and palatine/lingual bone cortices with periodontal probe.

The affected teeth were extracted with the least possible trauma, without damaging the bone cortices (Fig. 1. The integrity of the vestibular and lingual cortices was then checked using a periodontal probe (Fig. 1). All surgeries were “flapless” and in no case was a mucoperiosteal flap raised. In the test group, after extracting the teeth with periapical pathology, the alveolae underwent meticulous curettage and debridement to eliminate all granulation tissue. Alveolae were disinfected with 0.12% chlorhexidine-soaked gauzes (applied for one minute) and abundantly irrigated with physiological serum and 0.12% chlorhexidine to eliminate any remaining detritus from the alveolus.

**Figure 2:**
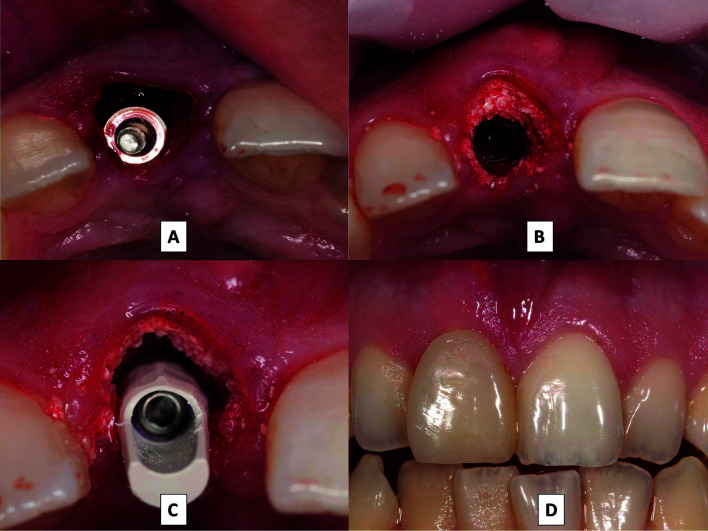
A: Slightly palatal immediate implant placement. B: Biomaterial placed in gap between implant and vestibular table. C: Provisional abutment.D: Immediate prosthetic provisional restoration.

Implant placement in both groups was performed following the usual drilling protocol indicated by the implant manufacturer, Biomet 3i (Zimmer Biomet, Palm Beach Garden, Florida, USA), applying insertion torque >35 N/cm2 in all cases. Implants were palatally positioned to leave more space between the vestibular bone cortex and the implant, afterwards filling this space with a bovine bone substitute, Endobone® (Zimmer Biomet, Palm Beach Garden, Florida, USA) (Fig. 2). Lastly, a provisional prosthetic restoration without occlusion (screwed resin single crowns with platform-switched provisional abutments) was screwed to the implant applying the torque recommended by the manufacturer (Fig. 2). All patients followed the same post-operative regime, continuing with the prescribed antibiotic treatment for a further 7 days after surgery, 600 mg Ibuprofen (three times a day for 4 days) and applications of 1.2% chlorhexidine gel in the surgical area (three times a day for 10 days).

- Follow-up

Six months after immediate implant placement with immediate prosthetic provisionalization, the definitive restoration was fabricated. Clinical and radiological variables were assessed 1-year after implant placement.To determine the success of the implants, the following criteria were considered: absence of peri-implantitis (changes in the level of the crestal bone in conjunction with bleeding on probing with or without concomitant deeping of peri-implant pockets and presence of pus), lack of mobitlity, absence of persistant pain or dysesthesia, and absence of continuous radiolucency around the implant.

- Data collection (clinical parameters)

The peri-implant clinical data registered were: a) bleeding on probing; b) buccal width of keratinized mucosa (KM) at the implant site; and c) mesial papillary, distal papillary and mid-facial recession (MPR, DPR and MFR). Digital clinical photographs were used to evaluate MPR, DPR and MFR. To evaluate these three variables, calibrated photographs were taken using a cephalostat to maximize reproducibility. Photographs were obtained with a 6.1-megapixel digital camera (Canon® EOS 70D; Canon®, Tokyo, Japan) with 100 mm macro-lenses with minimal focal distance. An initial photo was taken (day 0: placement of the provisional prosthetic restoration) and 1-year after implant placement. Measurements were taken at three different points on the implant’s vestibular surface and the crown: level with the mesial papilla, the distal papilla, and the tip. Image analysis software was used to take these measurements IMAGEJ version 1.46 (National Institute of Health, Maryland, USA). Lastly, probing depth (PD) was measured at six sites, three vestibular (mesial, central and distal) and three palatine/lingual (mesial, central and distal); using a manual periodontal probe, Hu-Friedy® CP 15 UNC (Hu-Friedy®, Chicago, IL, USA).

- Data collection (Radiographic parameters)

For evaluation of radiographic bone loss, a digital radiography system (RVG Model 5100, Kodak, Rochester, NY, USA) was used with Rinn-XCP support (DentsplyRinn, Elgin IL, USA). All radiographs were captured at 70 Kv, 8 mA with a focal distance of 30 cm. Mesial, distal and total crestal bone loss (mesial + distal / 2) (vertical distance from the implant shoulder to the first bone-to-implant contact IS-BIC) were measured using digital image analysis software, IMAGEJ version 1.46 (National Institute of Health, Maryland, USA).

- Statistical analysis

Data were analyzed using the SPSS version 20.0 statistical package (SPSS® Inc., Chicago, IL, USA). A descriptive study was made of each variable. The associations between the different qualitative variables were analyzed using Pearson’s chi-squared test. Student’s t-test for two independent samples was used in application to quantitative variables, in each case determining whether variances were homogeneous. Statistical significance was established as p≤0.05.

## Results

This prospective study recruited 100 patients (45 men and 55 women), with an average age of 48.19 ± 12.16 years. The sample was divided into two groups of 50 patients, a test group with periapical pathology, and a control group of 50 patients without periapical pathology. The success rate was 100% in both groups after 1-year implant placement.

When the homogeneity of the two groups was checked, they were found to be homogenous in terms of age, sex, smoking, alcohol consumption, oral hygiene (tooth brushing and full-mouth plaque score) and primary implant stability with mean values of 40 N/cm2 in both groups (Table 1).

The reasons dental extractions were performed could be divided into two groups: acute infection (failed endodontic treatment, active periapical periodontitis, granuloma and pus or infected fracture), and no infection (non-functional root, tooth fracture, tooth mobility, tooth ankyloses or orthodontic traction). Fifty patients presented acute infection and 50 patients no infection. Among those with acute infection, the most common motive for extraction was an infected fracture (n=21, 42%), other 15 patients (30%) for granuloma and pus and 14 patients (28%) for failed endodontic treatment. In this group, the presurgical examination using cone beam computed tomography showed that only 10 patients had a small loss of bone (located in buccal wall in all patients), which in none case was greater than 2 mm. While in the non-infected group the most common motive was the fracture of a non-infected tooth (n=28, 56%) and other 22 patients (44%) for non-functional root.

The localization and characteristics of the implants inserted are shown in Table 2. All the implants placed were Biomet 3i (Zimmer Biomet, Palm Beach Garden, Florida, USA). Most implants were placed in the upper maxilla (81%), both in the test group(76%) and the control group (86%); and in posterior regions (75%), positions 1.4 (15%) and 1.5 (15%) being the most frequent. The most commonly used implant length was 13 mm (33%), while the most common diameter was 4.00 mm (66%) (Table 2).

When the test group (with peri-apical pathology) was compared with the control group (without periapical pathology), the control group obtained better peri-implant clinical results for all variables except for the presence of keratinized gum and probe depth. However, no differences were found to be statistically significant (*p*>0.05) for any of the variables between the two groups (Table 3).

When radiographic bone loss was compared between the groups, test group suffered greater bone loss than control group (0.35 ± 0.51 and 0.15 ± 0.87, respectively), although without statistically significant difference (*p*=0.160) (Table 4).

**Table 1:**
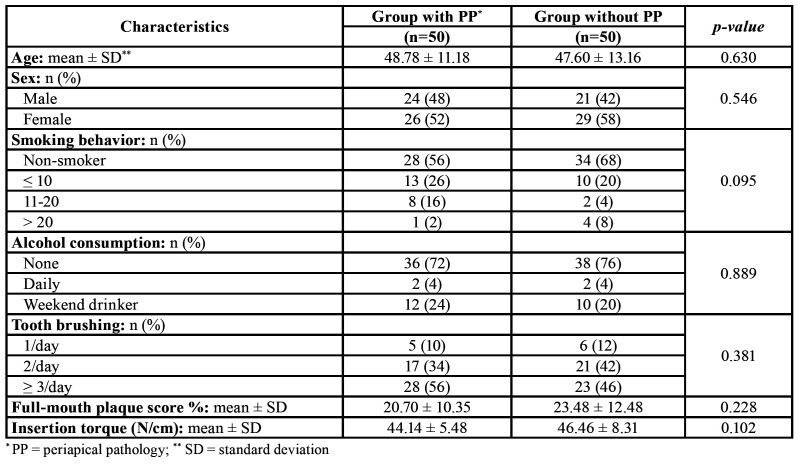
Homogeneity of study groups in terms of demographic characteristics, study level and habits (Student t-test and Pearson χ2).

**Table 2:**
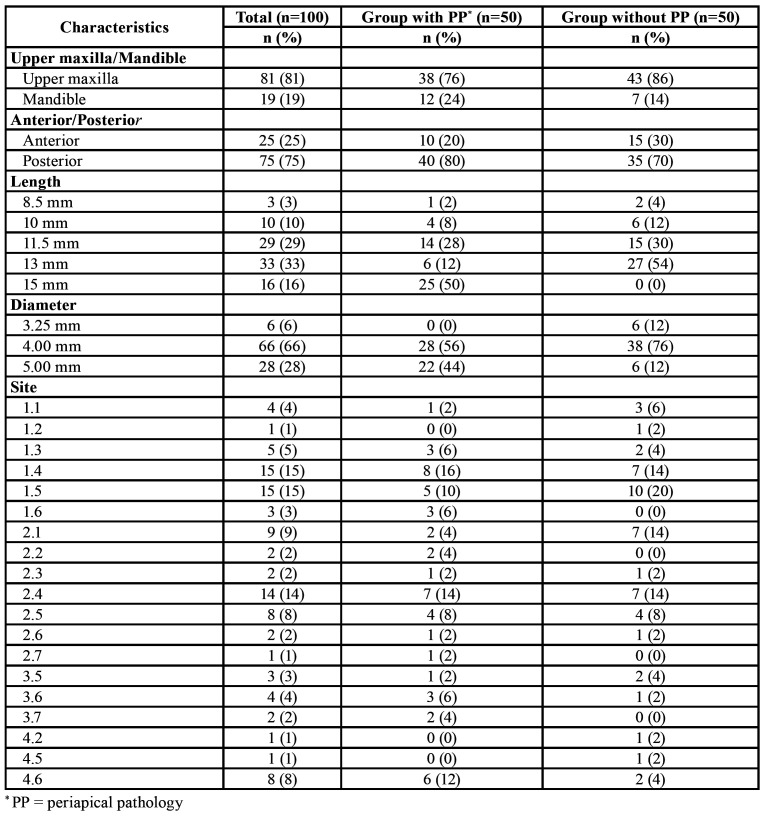
Implant distribution.

**Table 3:**
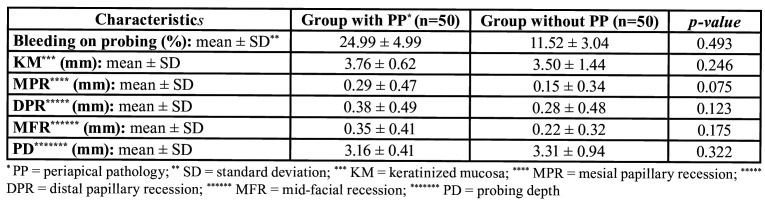
Comparison of clinical measurements between study groups (Student’s t-test).

**Table 4:**
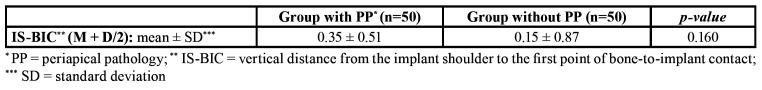
Comparison of radiographic bone loss between study groups (Student’s t-test).

## Discussion

This prospective study with 1-year follow-up, included 100 patients who each received a single immediate implant with immediate prosthetic provisionalization. The clinical and radiological results did not find statistically significant differences between patients presenting acute periapical pathology and those who did not.

These findings concur with previous animal studies, which have observed similar implant success rates for immediate implants placed in alveolae with and without periapical pathology (19-22). The present results also coincide with clinical trials conducted by other authors (23-27), who obtained excellent results with implants placed in post-extraction alveolae with previous periapical pathology, applying a rigorous protocol of curettage and debridement of the lesions, although these authors did not perform immediate prosthetic provisionalization. To obtain these good and predicTable results, all the authors agree (23-27) (as we do) that the most important thing is a good protocol of alveolus debridement and meticulous cleaning after tooth extraction: first, all granulation tissue should be carefully removed with curettage and debridement; second, alveolae should be disinfected with 0.12% chlorhexidine-soaked gauzes (applied for one minute); and finally, abundantly irrigation with physiological serum and 0.12% chlorhexidine is necessary to eliminate any remaining detritus from the alveolus.

In 2012, Jofre *et al* *et al*., (28) obtained excellent clinical results placing immediate implants with immediate provisionalization in alveolae with periapical pathology. The trial did not compare the outcomes with a control group. Implant placement surgery was performed raising mucoperiosteal flaps in almost half the sample.

The present study’s main limitation was the impossibility of comparing the results with other research, as this is the first time that a investigation of the viability of immediate implant placement in alveolae with periapical pathology has been conducted using the flapless technique and immediate provisionalization, also comparing the outcomes with a control group of the same size as the test group.

Lindeboom *et al* *et al*., (23), Siegenthaler *et al* *et al*., (24), and Blus *et al* *et al*., (27) carried out prospective controlled clinical trials in which immediate implants were placed in post-extraction alveolae in patients with periapical pathology, although these researchers did not perform immediate prosthetic provisionalization. Nor did Crespi *et al* *et al*., (25) and Truninger *et al* *et al*., (26) perform immediate provisionalization in their studies of implants placed in alveolae with periapical pathology; these two trials also obtained good clinical results over 3- and 4-year follow-ups respectively.

The present study recorded the reason for each extraction, finding that in the test group, all extractions were performed because of acute infection. Blus *et al* *et al*., (27) also placed immediate implants in alveolae with periapical pathology, reporting that 43.38% of the test sample presented acute infection, while 56.62% presented chronic infection.

Mean radiographic bone loss values did not show statistically significant differences between the test and control groups. Our results (0.35 ± 0.51 mm in the test group and 0.15 ±0.87 mm in the control group) differed from values obtained by Siegenthaler *et al* *et al*., (24) who also compared immediate implants placed in alveolae with and without periapical pathology, obtaining the following mean values: 1.5 ± 0.8 mm (test group) and 1.35 ± 0.95 mm (control group). Nevertheless, they did not find significant differences between the groups. The bone loss obtained by Truninger *et al* *et al*., (26) was 0.41 ± 0.49 mm in the test group and 0.12 ± 0.74 mm in the control group, less radiographic bone loss than observed by Crespi *et al* *et al*., (25) (0.79 ± 0.38 mm in the test group and 0.78 ± 0.38 mm in the control group). As in the present study, these researchers did not find statistically significant differences between the groups.

The present study suffered several limitations. Firstly, the sample sizes were not large, and secondly, a follow-up period of over 10 years is needed. Moreover, it was not possible to compare the results with any other study with the same surgical protocol and follow-up period.

In conclusion, after careful debridement of the extraction socket, immediate placement of implants with immediate prosthetic provisionalization at sites with acute periapical pathology can be a successful treatment modality for at least 1 year, with no disadvantages in clinical and radiological parameters compared with immediate implants placed in sites without periapical pathology.

## References

[1] Cornellini R, Cangini F, Covani U, Wilson TG Jr (2005). Immediate restoration of implants placed into fresh extraction shockets for single-tooth replacement: a prospective clinical study. Int J Periodontics Resotrative Dent.

[2] Esposito M, Grusovin MG, Willings M, Coulthard P, Worthington HV (2007). The effectiveness of immediate, early and conventional loading of dental implants: a Cochrane suystematic review of randomized controlled clinical trials. Int J Oral Maxillofac Implants.

[3] De Rouck T, Collys K, Cosyn J (2008). Single-tooth replacement in the anterior maxilla by means of immediate implantation and provisionalization: a review. Int J Oral Maxillofac Implants.

[4] Buser D, Halbritter S, Hart C, Bornstein M, Grütter L, Chappuls V (2009). Early implant placement with simultaneous guided bone regeneration following single-tooth extraction in the esthetic zone: 12-month results of a prospective study with 20 consecutive patients. J Periodontol.

[5] Chen ST, Wilson TG Jr, Hämmerle CH (2004). Immediate or early placement of implants following tooth extraction: review of biologic basis, clinical procedures, and outcomes. Int J Oral Maxillofac Implants.

[6] Schropp L, Isidor F, Kostopoulos L, Wenzel A (2005). Interproximal papilla levels following early versus delayed placement of single-tooth implants: a controlled clinical trial. Int J Oral Maxillofac Implants.

[7] Block MS (2011). Placement of implants into fresh molar sites: results of 35 cases. J Oral Maxillofac Surg.

[8] Cardaropoli D, Cardalopoli G (2008). Preservation of the alveolar ridge: a clinical and histologic study. Int J Periodontics Restorative Dent.

[9] Berglundh T, Lindhe J (1996). Dimension of the periimplant mucosa. Biological with revisited. J Clin Periodontol.

[10] Linkevicius T, Puisys A, Steigmann M, Vindasiute E, LInckeviciene L (2015). Influence of Vertical Soft Tissue Thickness on Crestal Bone Changes Around Implants with Platform Switching: A Comparative Clinical Study. Clin Implant Dent Relat Res.

[11] Misch CE, Perel ML, Wang HL (2008). Sammaino G, Galindo-Moreno P, Trisi P, et al. Implant success, survival, and failure: The International Congress of Oral Implantologists (ICOI) Pisa Consensus Conference. Implant Dent.

[12] Botticelli D, Berglundh T, Lindhe J (2004). Hard-tissue alterations following immediate implant placement in extraction sites. J Clin Periodontol.

[13] Canullo L, Iyrlaro G, Ianello G (2009). Double-blind randomized controlled trial study on post-extraction immediately restored implants using the switching platform concept: soft tissue response. Preliminary report. Clin Oral Implants Res.

[14] Hämmerle CH, Araújo MG, Simion M, Osteology Consensus Group 2011 (2012). Evidence-based knowledge on the biology and treatment of extraction sockets. Clin Oral Implants Res.

[15] Waasdorp JA, Evian CI, Mandracchia M (2010). Immediate placement of implants into infected sites: A systematic review of the literature. J Periodontol.

[16] Tolman DE, Keller EE (1991). Endosseous implant placement immediately following dental extraction and alveoloplasty: preliminary report with 6-year follow-up. Int J Oral Maxillofac Implants.

[17] Barzilay I (1993). Immediate implants: their current status. Int J Prosthodont.

[18] Quirynen M, Van Assche N, Botticelli D, Berglundh T (2007). How does the timing of implant placement to extraction affect outcome?. Int J Oral Maxillofac Implants.

[19] Novaes AB Jr, Vidigal Júnior GM, Novaes AB, Grisi MF, Polloni S, Rosa A (1998). Immediate implants placed into infected sites: a histomorphometric study in dogs. Int J Oral Maxillofac Implants.

[20] Novaes AB Jr, Marcaccini AM, Souza SL, Taba Jr, Grisi MF (2003). Immediate placement of implants into periodontally infected sites in dogs: a histomorphometric study of bone-implant contact. Int J Oral Maxillofac Implants.

[21] Marcaccini AM, Novaes AB Jr, Souza SL, Taba M Jr, Grisi MF (2003). Immediate placement of implants into periodontally infected sites in dogs. Part 2: a fluorescence microscopy study. Int J Oral Maxillofac Implants.

[22] Papalexiou V, Novaes AB Jr, Grisi MF, Souza SS, Taba M Jr, Kajiwara JK (2004). Influence of implant microstructure on dynamics of bone healing around immediate implants placed into periodontally infected sites. A confocal laser scanning microscopic study. Clin Oral Implants Res.

[23] Lindeboom JA, Tijook Y, Kroon FH (2006). Immediate placement of implants in periapical infected sites: a prospective randomized study in 50 patients. Oral Surg Oral Med Oral Pathol Oral Radiol Endod.

[24] Siegenthaler DW, Jung RE, Holderegger C, Roos M, Hämmerle CH (2007). Replacement of teeth exhibiting periapical pathology by immediate implants. A prospective, controlled clinical trial. Clin Oral Impl Res.

[25] Crespi R, Capparè P, Gherlone E (2010). Immediate loading of dental implants placed in periodontally infected and non-infected sites: a 4-year follow-up clinical study. J Periodontol.

[26] Truninger TC, Philipp AOH, Siegenthaler DW, Roos M, Hämmerle CH, Jung RE (2011). A prospective, controlled clinical trial evaluating the clinical and radiological outcome after 3 years of immediately placed implants in sockets exhibiting periapical pathology. Clin Oral Impl Res.

[27] Blus C, Szmukler-Moncler S, Khoury P, Orrù G (2015). Immediate implants laced in infected and noninfected sites after atraumatic tooth extraction and placement with ultrasonic bone surgery. Clin Implant Dent Relat Res.

[28] Jofre J, Valenzuela D, Quintana P, Asenjo-Lobos C (2012). Protocol for immediate implant replacement of infected teeth. Implant Dent.

[29] Von Elm E, Altman DG, Egger M, Pocock SJ, Gøtzsche PC, Vanderbroucke JP (2014). The Strengthening the Reporting of Observational Studies in Epidemiology (STROBE) Statement: guidelines for reporting observational studies. Int J Surg.

[30] O'Leary TJ, Drake RB, Naylor JE (1972). The plaque control record. J Periodontol.

